# Editorial: Advancing precision medicine in acute stroke care: personalized treatment strategies and outcomes

**DOI:** 10.3389/fmed.2025.1718309

**Published:** 2025-11-11

**Authors:** Mohamed F. Doheim, Nirav Bhatt, Alhamza R. Al-Bayati, Jean-Claude Baron, Joao Brainer Clares de Andrade

**Affiliations:** 1 University of Pittsburgh Medical Center (UPMC) Stroke Institute and Department of Neurology, University of Pittsburgh School of Medicine, Pittsburgh, PA, United States; 2 The Sainte-Anne Hospital Center, Paris, France; 3 Department of Health Informatics, Universidade Federal de São Paulo, São Paulo, Brazil; 4 Academic Research Organization, Einstein Hospital Israelita, São Paulo, Brazil

**Keywords:** precision medicine, stroke, neurology, outcomes - health care, stroke care

## Introduction

Precision medicine in acute stroke care has matured from a visionary concept into an operational framework. The articles in this Research Topic, spanning molecular biomarkers, imaging phenotypes, therapeutic optimization, neuro-recovery science, and health-system engineering, all converge on a single imperative of individualizing decisions at the speed and scale required by cerebrovascular emergencies. Below, we summarize the cross-cutting insights, highlighting how this body of work collectively reframes the concept of “time is brain” as “the right treatment for this brain now.”

## Biology that stratifies risk and reveals targets

Routine, low-cost laboratory tests can carry disproportionate prognostic value and a huge return on investment, presenting an opportunity for risk stratification and decoding prognosis. For instance, Wang et al. reported on a large ischemic stroke cohort of 11,405 patients and found that baseline alkaline phosphatase (ALP) was associated with 3-month and 1-year mortality, poor functional outcomes, and increased disability. Marked variability was noted among subtypes representing different etiologies. These results may suggest potential links between mineral metabolism, vascular calcification, and neurorepair while arguing for the validation of ALP as a triage signal for follow-up intensity. Similarly, a retrospective study of 200 patients with aneurysmal subarachnoid hemorrhage (aSAH) reported by Min et al. showed that, on postoperative day 7, the neutrophil count, neutrophil-to-lymphocyte ratio (NLR), systemic inflammatory response index (SIRI), and systemic immune-inflammation index (SII) were significantly higher in patients with poor outcomes vs. those with good outcomes. In multivariate analysis, cerebrospinal fluid (CSF) red blood cell (RBC) count on day 1 (≥177 × 10^9^/L; OR 7.227, 95% CI: 1.160–45.050, *P* = 0.034), surgical duration (≥169 min), Fisher grade (III–IV), hypertension, and infections were associated with poor outcomes. On day 7, a CSF RBC count (≥54 × 10^9^/L; OR 39.787, 95% CI: 6.799–232.836, *P* < 0.001) and an NLR (≥8.16; OR 6.362, 95% CI: 1.424–28.428, *P* = 0.015) remained independent predictors. NLR (*r* = 0.297, *P* = 0.007) and SIRI (*r* = 0.325, *P* = 0.003) correlated with CSF RBC count. Overall, elevated NLR and CSF RBC count were strongly associated with poor prognosis in aSAH. These findings suggest that neuroinflammation may be both a marker and a modifiable pathway.

Building on this idea, renal indices do not reflect a mere comorbid noise. In a cohort of 230 patients with acute large vessel occlusion (LVO) stroke, Rocha et al. showed that nearly one-third of the patients exhibited a fast progressor phenotype, characterized by higher serum creatinine, lower estimated glomerular filtration rate (eGFR), and substantially worse clinical outcomes. Elevated creatinine levels (≥1.2 mg/dl) independently predicted fast progression, poor 90-day functional recovery, and mortality, while reduced eGFR (< 60 ml/min/1.73 m^2^) was associated with fast progression but not with longer-term outcomes. These findings suggest that simple, routinely available creatinine-based biomarkers of renal dysfunction may serve as early indicators of aggressive infarct dynamics, helping clinicians identify high-risk patients during emergency evaluations and prioritize them for expedited endovascular therapy.

Two contributions extend this biological lens even further. First, a systematic review performed by Al-Jehani et al. positioned microRNAs as plausible tools for stratifying risk and forecasting outcomes in ischemic stroke, spanning signatures of endothelial dysfunction, inflammation, and thrombus biology. In contrast, a study of 317 patients of whole-blood viscosity (WBV) after thrombectomy reported by Thapa et al. found no association with discharge disability; however, this finding may be influenced by limitations in the formula used to estimate shear rates. While this report suggests that WBV alone does not directly impact prognosis, other determinants of blood viscosity may still meaningfully influence outcomes after thrombectomy and warrant further investigation.

Finally, an interpretable machine learning model incorporating the platelet distribution width-to-platelet count ratio (PPR) to predict hemorrhagic transformation (HT) after reperfusion was developed by Li, Lei, et al. Among AIS patients treated with IVT, six features—age, diabetes, malignancy, onset-to-treatment time, baseline NIHSS score, and PPR—were identified via LASSO regression. Logistic regression (LR) outperformed other models, achieving an AUC of 0.919, with accuracy, sensitivity, and specificity around 0.83. Feature importance ranked baseline NIHSS, diabetes, and PPR highest. The LR-based model offers a rapid, accurate tool to predict HT risk and support clinical decision-making in AIS patients receiving thrombolysis.

## Imaging phenotypes that individualize decisions

Several studies clarify which images matter when the stakes are highest. In a study by Yeo et al., including 325 consecutive patients with anterior circulation LVO, multiphase CTA collateral grade strongly discriminated 3-month outcomes among EVT candidates, reinforcing the importance of collateral-aware selection and expectation-setting beyond “time alone.” Li, Gao. et al., on the other hand, showed that high-resolution vessel-wall MRI features (intraplaque hemorrhage and normalized wall index) combined with clinical scores effectively predict recurrence risk in patients with high-risk, non-disabling ischemic cerebrovascular events, and the resulting nomogram offers a practical tool for identifying high-risk individuals.

Beyond occlusion-centric selection, covert substrate matters. Abdulsalam et al. reported that silent brain infarcts and leukoaraiosis co-occurred in middle-aged adults and were associated with each other in a cohort of 50 patients, arguing that the burden of small-vessel disease should inform cognitive surveillance and counseling after apparently “minor” strokes.

Two articles deepen physiologic precision. A prospective, serial-MRI study by Carbó et al. quantified impaired microvascular reperfusion (IMR) in one-quarter of “successfully reperfused” patients; larger IMR volumes correlated with worse early neurological status, highlighting evidence that tissue-level heterogeneity persists despite angiographic success and that IMR is a plausible target and imaging surrogate for adjuvant therapies. A radiomics approach reported by Yang et al. that fused DSC-PWI “critical-moment” features with conventional parameter maps achieved superior outcome prediction (AUC ~0.92), illustrating the value of richer spatiotemporal signatures. This approach has the potential to improve AIS prognosis assessment and aid clinicians in selecting optimal treatments.

Imaging also refines our ability to identify etiology. A meta-analysis by Xu et al. showed that cardiac CT angiography (CCTA) detects intracardiac thrombus in ~8% of AIS within 1 month, including many patients without documented AF, supporting the use of early CCTA when TEE is delayed or poorly tolerated, as it can accelerate secondary prevention.

## Optimizing reperfusion and adjuncts

Therapeutic precision spans drugs, devices, and dosing. A network meta-analysis by Sun et al. across randomized trials suggested that reteplase may increase the odds of an excellent 90-day outcome compared to alteplase. Tenecteplase at a dose of 0.25 mg/kg was found to be the most effective in achieving good outcomes with a similar safety profile, encouraging phenotype- and dose-specific trials. In an MRI-selected mild stroke (NIHSS ≤ 5) study reported by Li, Chen, et al., dual antiplatelet pretreatment plus low-dose rt-PA (0.6 mg/kg) yielded higher early neurological improvement and better 90-day function than standard-dose rt-PA or DAPT alone, without an increased risk of hemorrhage. This is hypothesis-generating evidence that a biology- and imaging-guided approach to tailoring doses may benefit mild phenotypes.

Adjunctive strategies are moving from theory to plausible practice. In a propensity-matched cohort by ElBassiouny et al., early cerebrolysin administration after thrombectomy for cardioembolic LVO was associated with greater independence and less hemorrhagic transformation—results that now demand randomized confirmation, ideally with IMR-type tissue metrics as mechanistic endpoints. For intracerebral hemorrhage, minimally invasive evacuation using a YL-1 hematoma crushing needle was associated with faster resolution and improved short-term function compared to conservative care in a retrospective series reported by Chen et al.. Their findings support MIS pathways where craniotomy risk or delay is prohibitive, pending controlled trials.

Posterior circulation epidemiology reminds us that case mix dominates. A U.S. National Inpatient Sample analysis conducted by Saha et al., focusing on vertebrobasilar occlusion (VBAO), showed that younger patients (18–64 years) tended to receive EVT more frequently than older patients, with no significant differences observed between sexes, likely reflecting selection and severity. Cancer-related stroke exemplifies uncertainty where precision is needed most. Kielkopf et al. showed that, in patients with active cancer and AIS, anticoagulation vs. antiplatelet therapy at discharge yielded similar adjusted risks for 1-year mortality and recurrent stroke, highlighting the need for randomized, biology-anchored selection.

## Systems that compress time and align care with phenotype

Operational precision is where biomarkers and images become minutes saved. A Hybrid Emergency Room System (HERS) reported by Kashiura et al. shaved ~30–40 min off door-to-puncture and door-to-recanalization times for EVT—an implementation signal that invites multicenter, phenotype-enriched evaluations where time sensitivity is greatest. Population-level lenses ensure that translation is equitable and context-aware. In Jordan, the 1-year stroke incidence in AF was 3.4%, with diabetes and prior stroke conferring ≈2.6-fold higher odds, as reported by Al-Shatanawi et al.. This can inform the tailoring of anticoagulation strategies to local comorbidity profiles. In China, Tang et al. reported that the burden of ischemic stroke attributable to high fasting plasma glucose increased in absolute terms from 1990 to 2019, with projections suggesting future decline under current trends, reinforcing glycemic control as a population lever for precision prevention.

## Recovery science that personalizes rehabilitation

Precision extends to neurorecovery. A systematic review of sensorimotor network (SMN) alterations across fMRI studies by Sahrizan et al. documented early disruptions with progressive reintegration through compensatory reorganization; lesion topology was found to modulate trajectories, advocating for network-aware rehabilitation that adapts to time since stroke and structural substrate.

## A practical synthesis for the bedside

Collectively, these studies outline a layered, workflow-embedded model:

1) Baseline biology, such as ALP, creatinine/eGFR, microRNAs, and inflammatory signals, flags trajectories (e.g., fast progression, poor repair) and informs hemodynamic goals, nephro-pharmacologic caution, and surveillance intensity.
2) Imaging phenotypes, such as collaterals, vessel-wall features, IMR, radiomics, and covert SVD, personalize EVT candidacy, adjuvant choices, and cognitive follow-up.
3) Etiologic precision, such as early CCTA to detect thrombus even without known AF, accelerates anticoagulation in selected patients.
4) Therapeutic tailoring, i.e., dose- and phenotype-aware thrombolysis, candidate neuroprotective adjuncts (e.g., cerebrolysin), and MIS for ICH, where feasible, should be tested with tissue-level surrogates (e.g., IMR) and patient-centered endpoints.
5) Operational precision, such as HERS-like infrastructures and collateral/fast-progressor-triggered fast tracks, must prove their ability to convert minutes into disability-free days, especially in the posterior circulation and in cancer-related phenotypes, where equipoise persists.

## Conclusion

This Research Topic offers a coherent, actionable blueprint for precision stroke care: measure what matters, when it matters, for the patient in front of you. By combining accessible biomarkers (e.g., ALP, renal indices, microRNAs), richer imaging phenotypes (e.g., collaterals, vessel-wall features, IMR, radiomics, covert SVD), optimized reperfusion and adjunct strategies (e.g., dose-tuned thrombolysis, neuroprotection, MIS), and systems engineering (e.g., HERS, phenotype-triggered pathways), the field is poised to transform variability into value. The next phase hinges on rigorous external validation, workflow-embedded trials, and equitable deployment so that personalization augments, rather than replaces, clinical judgment for every patient we serve.

